# Case Report of Unilateral Short‐Root Abnormality Disease of Multiple Roots

**DOI:** 10.1155/carm/1295291

**Published:** 2026-08-27

**Authors:** Zhihao Tang, Xiaodan Fang, Yuxin Yang, Xin Liu

**Affiliations:** ^1^ Xiang Ya Stomatology Hospital of Central South University, Changsha, Hunan, China

**Keywords:** differential diagnosis, facial asymmetry, mandible, multiple teeth, short root anomaly, unilateral

## Abstract

Short root anomaly (SRA) is a rare developmental disturbance characterized by markedly shortened and blunted roots with a root‐to‐crown ratio below 1, while the crown morphology and periodontal structures remain unaffected. Most reported cases show bilateral symmetric involvement of maxillary anterior teeth. This paper presents a unique case of unilateral multiple SRA affecting mandibular canine, premolars, and molars combined with facial asymmetry in a 22‐year‐old male patient. No active treatment was performed except standardized oral hygiene instructions and regular follow‐up. This case expands the phenotypic spectrum of SRA and highlights the importance of comprehensive differential diagnosis.

## 1. Introduction

Short root anomaly (SRA) is a dental developmental malformation defined by blunted and short roots with a root‐to‐crown ratio < 1, whereas the dental crowns and periodontal tissues remain intact [1]. Previous epidemiologic and clinical studies have demonstrated that SRA predominantly affects bilateral maxillary central incisors, followed by maxillary premolars and lateral incisors. Typical radiographic features include normal coronal structure, normal periodontal ligament space, and conical, shortened roots [2]. Cases involving unilateral mandibular canines, premolars, and molars combined with facial asymmetry are extremely rare. The etiology of SRA is multifactorial, including genetic factors, systemic diseases, local trauma, infection, and iatrogenic interventions. However, the exact mechanism of unilateral multiple SRA remains unclear. Due to its low prevalence and atypical presentation, such cases are easily misdiagnosed as root resorption or other developmental anomalies.

This study reports a rare case of idiopathic unilateral multiple SRA involving the right mandibular posterior teeth in a 22‐year‐old male patient, accompanied by facial asymmetry and mandibular hypoplasia. The clinical manifestations, radiographic characteristics, possible etiology, molecular basis, differential diagnosis, and management strategy are comprehensively discussed.

## 2. Case Presentation

A 22‐year‐old male patient was referred to Xiangya Stomatology Hospital due to cold and heat sensitivity in the left maxillary posterior teeth for one week. He denied hypertension, heart disease, drug allergies, systemic diseases, or genetic disorders. His parents were nonconsanguineous and healthy, with no family history of dental anomalies. Detailed inquiry confirmed no history of trauma, severe infection, orthodontic treatment, or radiation therapy during the deciduous or mixed dentition period.

**FIGURE 1:**
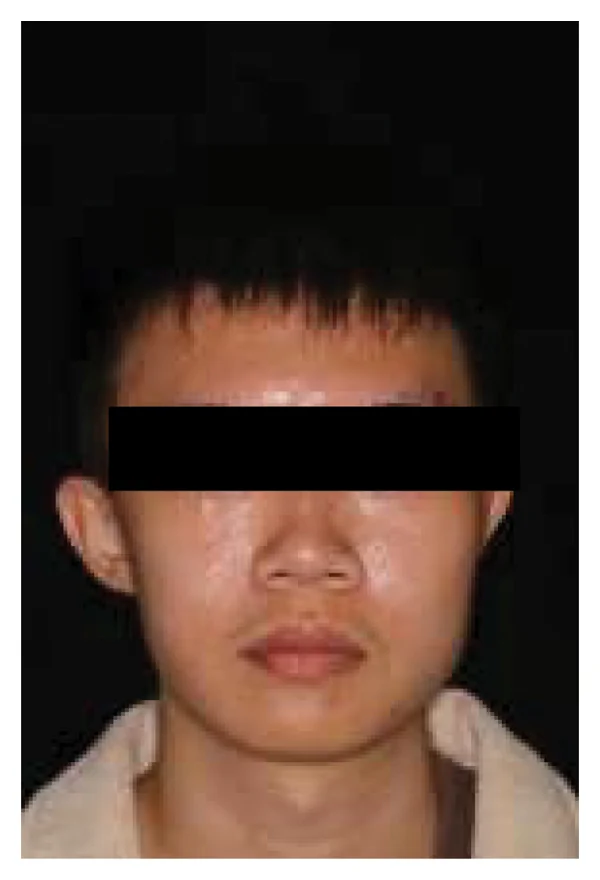
Facial asymmetry: pogonion on the right, lower left side more full than right side.

**FIGURE 2:**
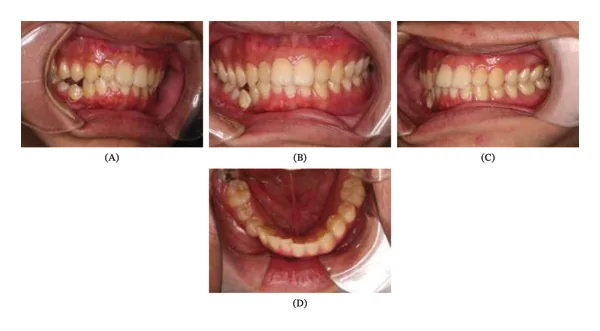
Intraoral examination of the occlusal relationship and caries. (A) On the right side, the premolars demonstrate an open bite, while the molars display a disto‐occlusal relationship. (B) Misalignment of the upper and lower midlines. (C) On the left side, the canines are in a tip‐to‐tip relationship, while the molars exhibit a mesio‐occlusal relationship. (D) Pit and fissure caries are observed on teeth 46 and 47.

Physical examination: The patient had normal nutritional status, skeletal development, and intelligence. Facial asymmetry was prominent: the facial midline shifted to the right, the right side of the lower face was underdeveloped, and the chin point deviated to the right (Figure 1). Mouth opening was normal but slightly deviated to the right. Intraoral examination revealed pit‐and‐fissure caries in teeth 26, 27, 46, and 47 with normal pulp vitality. Teeth 43–47 showed no mobility or percussion pain. The right molars and canines presented disto‐occlusion. The right premolars had no occlusal contact, with supraeruption of maxillary antagonists and a lower occlusal plane (Figure 2).

**FIGURE 3:**
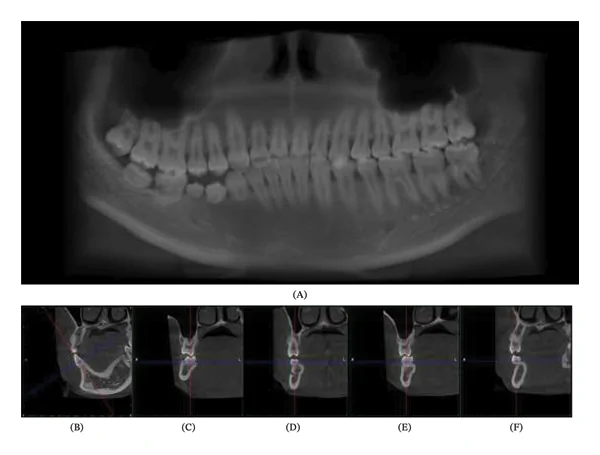
Cone‐beam CT examination. (A) CT cross section showing the development of whole dentition; (B)coronal surface of the right lower cusp; (C) coronal surface of the first right premolar; (D) coronal surface of the second right premolar; (E) coronal surface of the first right molar; and (F) coronal surface of the second right molar.

Radiographic examination: Cone‐beam computed tomography (CBCT) showed normal crown morphology, pulp chambers, and periodontal support in teeth 43–47 (Figure 3). All affected teeth exhibited significantly shortened roots with blunted apices and root‐to‐crown ratio < 1 (Table 1). No root resorption, periapical lesions, or alveolar bone abnormalities were detected.

**TABLE 1:** The lengths of anatomical crowns and roots of each right lower cusp to right second molars measured by CT, with a root‐to‐crown ratio less than 1.

Tooth position	Length of anatomical crown (mm)	Length of anatomical root (mm)	Root‐to‐crown ratio (root length:crown length)
43	6.72	2.62	0.39
44	6.80	2.40	0.35
45	4.00	1.81	0.45
46	5.80	4.60	0.79
47	4.80	2.20	0.46

Treatment and follow‐up: Since the affected teeth were asymptomatic and vital, no active treatment was performed. The patient received standardized oral hygiene instructions and was scheduled for follow‐up every 6 months. Annual radiographic re‐examination was recommended to monitor the root length, periodontal status, and alveolar bone. If mobility or clinical symptoms occur in the future, extraction and prosthetic rehabilitation would be considered. Up to the date of manuscript submission, the patient completed the first scheduled 6‐month follow‐up, with favorable and stable findings. Unfortunately, the subsequent planned follow‐up could not be conducted on time due to the patient’s off‐province academic arrangements.

## 3. Methods

This case report has been written according to Preferred Reporting Items for Case reports in Endodontics (PRICE) 2020 guidelines (Nagendrababu et al. 2020, doi: 10.1111/iej.13285).

All CBCT examinations were performed with standardized parameters (90 kV, 8 mA; field of view: 10 × 8 cm; voxel size: 0.25 mm). Two experienced oral radiologists independently measured crown height and root length of teeth 43–47 using the built‐in software tools, and the root‐to‐crown ratio was calculated to meet the SRA diagnostic cutoff (ratio < 1). Disagreements were resolved by consensus review to ensure reliability. All measured data are summarized in Table 1.

## 4. Discussion

Tooth root development is a sophisticated biological process governed by precise epithelial‐mesenchymal interactions and sequential molecular signaling cascades [3]. Any disruption during the critical window of root elongation may lead to developmental anomalies such as SRA [4]. The present case exhibited a rare phenotype of unilateral multiple SRA involving the mandibular canine, premolars, and molars, combined with obvious facial asymmetry and right mandibular hypoplasia, which is distinctly different from conventional SRA cases reported in previous literature. This rare combination expands the clinical spectrum of SRA and warrants in‐depth discussion regarding etiology, differential diagnosis, pathogenesis, and long‐term management.

### 4.1. Etiology and Pathogenetic Considerations

The etiology of SRA remains multifactorial and not fully elucidated. Proposed causes include genetic predisposition, systemic disorders, local trauma, infection, iatrogenic interventions, and idiopathic factors [5]. In this case, comprehensive history taking revealed no family history of dental anomalies, no consanguinity, no systemic diseases, no orthodontic treatment, no radiation exposure, and no definite history of severe trauma or infection during the deciduous or mixed dentition stages. These findings exclude most inherited and acquired etiologies.

Given the unilateral, localized distribution restricted to the right mandible, we hypothesize that the pathogenesis is most likely attributed to local, transient, and occult disturbances during root development. Subclinical local inflammation or minor mechanical insult during the early developmental phase of permanent tooth roots may have interrupted the normal proliferation and differentiation of Hertwig’s epithelial root sheath (HERS), thereby arresting root elongation without affecting crown morphogenesis [6]. Because such mild local injuries are often asymptomatic and unnoticeable, they are rarely documented in clinical history. Although genetic mosaicism cannot be entirely excluded, the absence of familial occurrence and the strictly unilateral pattern favor a sporadic, localized developmental disturbance rather than a Mendelian genetic disorder.

### 4.2. Molecular Regulatory Mechanisms

**FIGURE 4:**
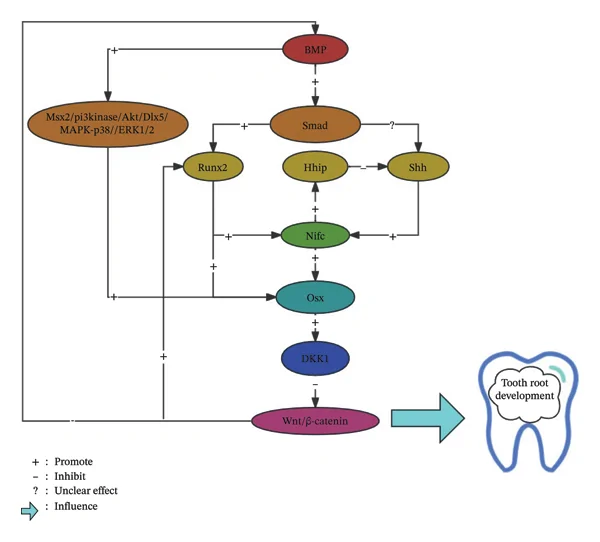
The regulatory signaling network in tooth root development. BMP signaling induces Osx activity through both Runx2‐dependent and Runx2‐independent pathways. The former involves the participation of the Smad mediator proteins, which act directly or indirectly through Nfic on Osx via the BMP/Smad/Runx2 axis. These pathways include Msx2, PI3K/Akt, Dlx5, MAPK‐p38, and ERK1/2, which regulate the downstream factor Osx. Osx, in turn, directly promotes DKK1 expression to inhibit the Wnt/β‐catenin signaling pathway. Simultaneously, the Wnt/β‐catenin pathway acts as a major inhibitor downstream of the BMP/Smad/Runx2/Nfic/Osx axis, downregulating BMP signaling and promoting Runx2 expression through a feedback loop.

Recent molecular studies have identified several core signaling pathways essential for tooth root formation, including Nfic, Shh, BMP, TGF‐β, and Wnt/β‐catenin cascades [7] (Figure 4). These pathways modulate odontoblast differentiation, root elongation, and dentin formation. Among them, Nfic acts as a master transcription factor for root development; its deficiency leads to impaired odontoblast polarity and short roots. Shh signaling supports the proliferation of mesenchymal cells surrounding HERS [8]. Wnt/β‐catenin signaling must be tightly regulated: both excessive activation and insufficient activity result in defective root development [9]. In the present case, transient local disruption of these pathways at the early root‐forming stage could explain the localized root shortening. This molecular interpretation supports the clinical observation that crowns and periodontal tissues remained intact, while root elongation was selectively arrested.

Based on molecular regulatory mechanisms, we further put forward one plausible pathogenetic hypothesis for this idiopathic unilateral multiple SRA. Transient subclinical microinjury during root development disrupts core signaling molecules and triggers abnormal pathway activation. This dysregulation suppresses proliferation and differentiation of HERS, arresting root elongation while crown formation remains intact. Future multicenter case accumulation combined with genetic analysis is necessary to validate the above conjectures.

### 4.3. Differential Diagnosis

Multiple developmental disorders, pathological lesions, and iatrogenic complications can exhibit similar radiographic features. Differential diagnosis should rely on medical history, lesion distribution, imaging manifestations, and concomitant anatomical changes. Classic SRA typically presents as bilateral symmetric root shortening confined to maxillary anterior teeth, with normal crowns and periodontal tissues and nonprogressive lesions [10]. Our case, involving multiple unilateral mandibular teeth, is a rare variant distinct from typical SRA. Regional odontodysplasia (ghost teeth) is featured by severe enamel‐dentin hypoplasia, hypomineralization, enlarged pulp chambers, and typical ghost‐like radiolucency [11], all of which were not observed in this patient. Pathological root resorption (external or internal) is a progressive condition induced by inflammation, trauma or orthodontic force, accompanied by root lacunar defects or widened root canals [12], and no such signs were identified here. Hemifacial microsomia manifests as severe unilateral craniofacial hypoplasia, frequently accompanied by ear malformations, tooth agenesis, or microdontia [13]. It does not cause isolated root shortening with intact crowns and periodontium. Isolated mandibular asymmetry leads to facial midline deviation and occlusal disharmony but presents no root malformations [14]. Iatrogenic root shortening is mainly caused by orthodontic treatment or dental surgery [15], and the patient had no relevant medical history. After excluding all above conditions based on the unilateral lesion distribution, intact crown, and periodontal tissues, as well as nonprogressive characteristics, we confirmed the diagnosis of idiopathic unilateral multiple SRA. The differential diagnosis is intuitively presented in Table 2.

**TABLE 2:** Differential diagnosis of diseases with shortened tooth roots.

Disease	Main manifestations	Exclusion basis
Classic SRA	Bilateral symmetric root shortening, mainly in maxillary anterior teeth	Lesions located at unilateral mandibular teeth
Regional odontodysplasia	Enamel/dentin hypoplasia, enlarged pulp chambers, ghost‐like radiolucency	Normal dental hard tissues and pulp
Pathological root resorption	Progressive root defects, related to trauma or inflammation	No resorption lesions or predisposing factors
Hemifacial microsomia	Severe facial hypoplasia, often with ear or tooth malformations	Only mild mandibular hypoplasia, no other malformations
Isolated mandibular asymmetry	Facial deviation and occlusal disorder, normal tooth roots	Obvious root shortening exists
Iatrogenic root shortening	Caused by orthodontic or surgical treatment	No relevant treatment history

### 4.4. Facial Asymmetry and Mandibular Hypoplasia

A distinctive feature of this case is the presence of facial asymmetry and right mandibular hypoplasia associated with localized SRA. The facial midline shifted to the right, the right hemiface was underdeveloped, and the chin deviated accordingly.

We propose two possible explanations: (1) Common developmental field defect: The same local disturbance that impaired tooth root development may also have mildly affected mandibular growth. (2) Secondary functional adaptation: Reduced vertical height and premature root arrest may have altered local occlusion and subsequently influenced symmetric jaw growth [16]^.^

### 4.5. Clinical Implications and Long‐Term Management

SRA is generally asymptomatic and nonprogressive; thus, an observational and conservative strategy is recommended. No active treatment is required for stable, asymptomatic teeth [17]. Key clinical messages include regular follow‐up every 6 months, annual radiographic monitoring, strict oral hygiene to prevent caries and periodontitis, avoidance of excessive occlusal force or traumatic prosthetic procedures, and extraction and prosthetic rehabilitation only if severe mobility or symptoms occur.

### 4.6. Limitations

Several limitations of the present report need to be addressed. Primarily, case reports are constrained by small sample size, and the unique phenotype of this patient cannot represent the overall characteristics of atypical SRA. Owing to clinical conditions and patient willingness, we did not conduct genetic screening or histological examination, which hinders the exploration of the molecular and genetic basis of this localized developmental defect. Moreover, the current follow‐up period is only short term, and the long‐term stability of root structure, occlusal function, and facial symmetry remains unknown. Multicenter case collection, combined with genetic and functional studies, will help to better reveal the etiology, clinical features, and management principles of rare SRA variants.

## 5. Conclusion

This atypical case describes unilateral multiple SRA of mandibular posterior teeth accompanied by facial asymmetry. Combined clinical and imaging evaluation together with systematic differential diagnosis facilitates reliable diagnosis. Conservative intervention and prolonged follow‐up are the mainstay of treatment. Our findings broaden the known phenotypic diversity of SRA and aid its clinical diagnosis and treatment.

## Funding

No funding was received for this manuscript.

## Ethics Statement

This retrospective case report utilizes fully anonymized clinical data and radiographic records without bringing any harm to the patient, and no sensitive personal information or commercial interests are involved. According to the relevant provisions stipulated in Article 32 of the Ethical Review Measures for Life Science and Medical Research Involving Humans (https://www.gov.cn/zhengce/zhengceku/2023-02/28/content_5743658.htm), this study meets the criteria for ethics review exemption. We have consulted the Institutional Review Board of Xiangya Stomatological Hospital, Central South University, and confirmed that formal ethical review is waived for this work. Written informed consent was voluntarily obtained from the patient prior to manuscript submission and publication. The patient was fully informed about the publication contents including clinical records and CBCT images. All identifiable personal information was thoroughly anonymized in the manuscript and figures to strictly protect patient confidentiality.

## Conflicts of Interest

The authors declare no conflicts of interest.

## Data Availability

The data that support the findings of this study are available on request from the corresponding author. The data are not publicly available due to privacy or ethical restrictions.

## References

[1] Trimeridou A. S. , Arhakis A. , and Arapostathis K. , Presentation of a Case of Short Root Anomaly in an 11-Year-Old Child, Case Reports in Dentistry. (2023) 2023, no. 1, 10.1155/2023/1766133. PMC9833915 36643592

[2] Apajalahti S. , Hölttä P. , Turtola L. , and Pirinen S. , Prevalence of Short-Root Anomaly in Healthy Young Adults, Acta Odontologica Scandinavica. (January 2002) 60, no. 1, 56–59, 10.1080/000163502753472014. 11902614

[3] Liu C. , Guo H. , Shi C. , and Sun H. , BMP Signaling in the Development and Regeneration of Tooth Roots: From Mechanisms to Applications, Frontiers in Cell and Developmental Biology. (September 2023) 11, 10.3389/fcell.2023.1272201. PMC10540449 37779895

[4] Lamani E. , Feinberg K. B. , and Kau C. H. , Short Root Anomaly—A Potential “Landmine” for Orthodontic and Orthognathic Surgery Treatment of Patients, Ann Maxillofac Surg. (2017) 7, no. 2, 296–299, 10.4103/ams.ams_128_16. 29264302 PMC5717911

[5] Sagawa Y. , Ogawa T. , Matsuyama Y. et al., Association Between Smoking During Pregnancy and Short Root Anomaly in Offspring, International Journal of Environmental Research and Public Health. (November 2021) 18, no. 21, 10.3390/ijerph182111662. PMC8582870 34770175

[6] Rao P. , Jing J. , Fan Y. , and Zhou C. , Spatiotemporal Cellular Dynamics and Molecular Regulation of Tooth Root Ontogeny, International Journal of Oral Science. (November 2023) 15, no. 1, 10.1038/s41368-023-00258-9. PMC10673972 38001110

[7] Sun K. , Yu M. , Wang J. et al., A Wnt10a-Notch Signaling Axis Controls Hertwig’s Epithelial Root Sheath Cell Behaviors During Root Furcation Patterning, International Journal of Oral Science. (March 2024) 16, no. 1, 10.1038/s41368-024-00288-x. PMC10937922 38480698

[8] Liu Y. , Feng J. , Li J. , Zhao H. , Ho T. V. , and Chai Y. , An Nfic-Hedgehog Signaling Cascade Regulates Tooth Root Development, Development. (October 2015) 142, no. 19, 3374–3382. 26293299 10.1242/dev.127068 PMC4631759

[9] Yu M. , Jiang Z. , Wang Y. , Xi Y. , and Yang G. , Molecular Mechanisms for Short Root Anomaly, Oral Diseases. (March 2021) 27, no. 2, 142–150, 10.1111/odi.13266. 31883171

[10] Lind V. , Short Root Anomaly, Scandinavian Journal of Dental Research. (1972) 80, no. 2, 85–93, 10.1111/j.1600-0722.1972.tb00268.x. 4505388

[11] Star J. M. , Jordan R. C. , and Stewart R. E. , PHACES Syndrome and Multi-Regional Odontodysplasia: A Case Report, Journal of Clinical Pediatric Dentistry. (May 2024) 48, no. 3, 166–170, 10.22514/jocpd.2024.070. 38755995

[12] Patel S. , Saberi N. , Pimental T. , and Teng P. H. , Present Status and Future Directions: Root Resorption, International Endodontic Journal. (October 2022) 55, no. Suppl 4, 892–921, 10.1111/iej.13715. 35229320 PMC9790676

[13] Luo S. , Sun H. , Bian Q. , Liu Z. , and Wang X. , The Etiology, Clinical Features, and Treatment Options of Hemifacial Microsomia, Oral Diseases. (September 2023) 29, no. 6, 2449–2462, 10.1111/odi.14508. 36648381

[14] Atiba P. M. , Omotoso B. R. , Madaree A. , and Lazarus L. , Hemifacial Microsomia: A Scoping Review on Progressive Facial Asymmetry due to Mandibular Deformity, Oral and Maxillofacial Surgery. (December 2024) 28, no. 4, 1441–1455, 10.1007/s10006-024-01276-5. 38954312 PMC11480165

[15] Pizzo G. , Licata M. E. , Guiglia R. , and Giuliana G. , Root Resorption and Orthodontic Treatment. Review of the Literature, Minerva Stomatologica. (2007) 56, no. 1-2, 31–44. 17287705

[16] Kim H. J. , Noh H. K. , and Park H. S. , Mandibular Asymmetry Types and Differences in Dental Compensations of Class III Patients Analyzed With Cone-Beam Computed Tomography, The Angle Orthodontist. (November 2023) 93, no. 6, 695–705, 10.2319/013023-73.1. 37407513 PMC10633797

[17] Rey D. , Smit R. M. , and Gamboa L. , Orthodontic Treatment in Patient With Idiopathic Root Resorption: A Case Report, Dental Press J Orthod. (2015) 20, no. 1, 108–117, 10.1590/2176-9451.20.1.108-117.oar. PMC4373023 25741832

